# Preliminary evaluation of a lemongrass-based nanoparticle gel for antibacterial control of *Enterococcus faecalis*: an *in vitro* study

**DOI:** 10.2340/biid.v13.46007

**Published:** 2026-05-19

**Authors:** Luedyna Rayane Rodrigues Leite, Maria Olívia da Costa, Álvaro de Souza Wanderley, Giovana Soriano Neiva, Carolina de Albuquerque Lima Duarte, Pedro Henrique Sette-de-Souza, Marlos Barbosa-Ribeiro

**Affiliations:** a School of Dentistry of Arcoverde, University of Pernambuco, Arcoverde, Brazil; b Postgraduate Program in Dentistry, School of Dentistry of Pernambuco, University of Pernambuco, Recife, Brazil

**Keywords:** *Cymbopogon citratus*, *Enterococcus faecalis*, nanoparticle, antimicrobial activity, medicinal plant

## Abstract

**Background:**

The search for biocompatible antimicrobial agents in endodontics has driven interest in phytotherapeutic compounds, including *Cymbopogon citratus* (lemongrass). This study aimed to perform a preliminary evaluation of the antibacterial potential of a lemongrass-based nanoparticle gel against *Enterococcus faecalis*.

**Materials and methods:**

Lemongrass leaves were collected, authenticated, and processed using different extraction methods. The most effective extract was selected for green synthesis of silver nanoparticles and incorporated into a 1% hydroxyethyl cellulose gel. Antibacterial activity against *E. faecalis* (ATCC 29212) was assessed by colony-forming unit (CFU/mL) quantification, agar well diffusion, and minimum inhibitory concentration (MIC) determination using resazurin. A 2.5% sodium hypochlorite (NaOCl) gel and 2% chlorhexidine (CHX) digluconate gel were used as positive controls, and a vehicle gel served as the negative control. Data were analyzed using Student’s *t*-test and the Wilcoxon test (α = 0.05).

**Results:**

The nanoparticulate lemongrass gel demonstrated significantly greater antibacterial activity than the non-nanoparticulated extract (*p* < 0.05), showing higher CFU/mL reduction and larger inhibition zones. The nanoparticle formulation maintained inhibitory activity at lower concentrations (MIC 50%) compared with the natural extract. Antibacterial performance of the nanoparticulate gel was comparable to CHX and superior to NaOCl in CFU reduction, while inhibition zones were similar to both controls.

**Conclusions:**

Nanoparticle incorporation significantly enhances the antibacterial efficacy of *C. citratus* against *E. faecalis*. The lemongrass-based nanoparticulate gel represents a promising alternative for use as a chemical auxiliary substance in endodontic treatment.

**HIGHLIGHTS**

- Lemongrass-based nanoparticles significantly enhance antibacterial activity against *Enterococcus faecalis*.
- Nanoparticulate gel formulation improves bacterial reduction and inhibition compared with the non-nanoparticulated extract.
- Lemongrass nanoparticulate gel is a promising biocompatible and cost-effective alternative for endodontic disinfection.
- The nanoparticulated *C. citratus* formulation showed preliminary in vitro antibacterial activity against *E. faecalis*, supporting further studies using endodontic strains and biofilm models.

## Introduction

Endodontic treatment involves the elimination of bacteria and neutralization of metabolic by-products within the root canal system. Successful outcomes require thorough cleaning, disinfection, shaping, three‑dimensional obturation of the root canal system, and subsequent coronal sealing [1, 2].

Chemical auxiliary substances (CHAS) used during chemomechanical preparation (CMP) should ideally possess antimicrobial activity, lubrication to ease instrumentation, tissue-dissolving capacity, chelating behavior, and biocompatibility [3]. Sodium hypochlorite (NaOCl, 1–6%) remains the gold standard in endodontic irrigation due to its tissue dissolution and antibacterial effects, but its high cytotoxicity poses risks of periapical tissue damage and mucosal contact may result in tissue necrosis [4]. On the other hand, chlorhexidine digluconate 2% (CHX) is considered a less toxic alternative, offering sustained antimicrobial action and lubricating properties when formulated as a gel although it lacks tissue dissolution capability [5, 6].

*Cymbopogon citratus* (Lemongrass), a member of the Poaceae family, is rich in bioactive compounds including citral, geraniol, terpenoids, flavonoids, phenolics, saponins, and tannins, which contribute to its antimicrobial, antioxidant, and anti-inflammatory properties [7]. It is widely used in traditional medicine and has demonstrated activity against oral pathogens such as *Enterococcus faecalis*, supporting its potential as an alternative or adjunct to conventional chemical irrigants [8–10].

The antimicrobial potential of *C. citratus* makes it a potential candidate pending further validation or adjunct to traditional chemical irrigants in root canal therapy [10]. Furthermore, its wide geographic distribution, ease of cultivation, and popular acceptance support its practical application in therapeutic contexts [11]. However, the persistence of *E. faecalis* has been reported in some cases of secondary or persistent endodontic infections, representing a relevant microbiological challenge for treatment [12, 13]. This microorganism is frequently isolated from secondary/persistent infections and exhibits a remarkable ability to survive harsh conditions within the root canal system, including resistance to starvation, high pH, and conventional mechanical and chemical disinfection methods [12]. Therefore, investigating natural products such as *C. citratus* against *E. faecalis* may provide valuable insights into more biocompatible and effective strategies for root canal disinfection.

Recent studies have demonstrated that nanocarrier systems (e.g. silver, polymeric, and lipid nanoparticles) can enhance the stability, bioavailability, and antimicrobial efficacy of phytochemicals, enabling controlled release and improved delivery to target sites [14, 15]. In dentistry, plant extract–loaded nanoparticles, particularly silver- and polymeric-based formulations, have shown promise in managing oral pathogens and disrupting biofilms [16, 17]. Among these systems, silver nanoparticles have received particular attention due to their well-documented broad-spectrum antimicrobial activity, which results from mechanisms such as membrane disruption, generation of reactive oxygen species, and interaction with bacterial proteins and DNA [17, 18].

Therefore, this study aimed to conduct a preliminary investigation of the antibacterial potential of a gel formulation containing silver nanoparticles synthesized from *C. citratus* extracts. Initially, different extraction methods were assessed to identify the most promising in terms of antimicrobial potential. Based on this, the nanoparticulate extract was produced, incorporated into a gel containing 1% hydroxyethyl cellulose and tested against *E. faecalis*. This exploratory approach focuses primarily on evaluating the biological activity of the formulation, representing an initial step toward the development of sustainable phytopharmaceuticals with potential application in endodontic disinfection protocols.

## Materials and methods

This is an in vitro experimental and analytical laboratory investigation designed to generate novel data with potential implications for improving health-related outcomes and contributing to the development of therapeutic products. All procedures were conducted at the Laboratório Multiusuário de Biotecnologia do Sertão Pernambucano (BIOSPE), School of Dentistry of Arcoverde, University of Pernambuco.

### Ethical considerations

This study did not involve human or animal subjects. Therefore, per Resolution 466/2012 of the Brazilian National Health Council (CNS), ethics committee approval was not required.

### Botanical collection and authentication

Fresh leaves of lemongrass were collected from rural areas in the municipality of Arcoverde, located in the semi-arid region of Pernambuco State, Brazil. Botanical identification was performed by specialists at the Herbarium Parque das Dunas (RN), where a voucher specimen was deposited (reference: RN7615). The research was duly registered in the National System for the Management of Genetic Heritage and Associated Traditional Knowledge (SisGen), under the Brazilian Ministry of the Environment, with registration number A75757E.

### Stage 1 – Preliminary study: extraction methods and phytopreparation

#### Extraction procedures

Ultrasound-assisted extraction

Dried *C. citratus* leaves were oven dried at 45°C (forced-air circulation) until mass stabilization (measured daily), followed by mechanical milling into fine powder (10 mesh). For each 1 g of powdered material, 10 mL of solvent was used (distilled water for aqueous extract, 100% ethanol for ethanolic extract, and 70% ethanol for hydroalcoholic extract). The mixture was placed in a sealed Erlenmeyer flask and subjected to ultrasonic bath (37°C for 60 minutes). The extracts were subsequently vacuum filtered and evaporated to dryness in a hot-air oven until the solvent became clear. Yields were calculated based on post-drying mass.

Orbital shaker-assisted extraction

An alternative method was also evaluated, adapted from Do Nascimento et al. [19]. Here, 30 g of powdered leaves were immersed in 72 mL of 100% ethanol in an Erlenmeyer flask and subjected to orbital agitation (IST3075R incubated shaker, Lab Companion) at 45°C and 150 rpm for 24 hours. The supernatant was collected by pipetting and the residual plant mass was subjected to a second 24-hour maceration (static) in an additional 72 mL of ethanol. Both fractions were combined, filtered, and evaporated. Extract yield was again calculated from dry mass.

#### pH determination

Extracts were diluted in sterile distilled water at concentrations of 100 mg/mL and 200 mg/mL, and pH was measured using non-bleeding MColorpHast™ indicator strips.

### Stage 2 – Formulation and antimicrobial evaluation of optimized extract

Based on preliminary results, the ethanolic extract obtained via shaker-assisted extraction was selected for nanoparticle synthesis and antimicrobial testing.

#### Synthesis of silver nanoparticles (AgNPs)

Silver nanoparticles were synthesized following plant-mediated green synthesis approaches previously described in the literature [18]. For every 10 mL of ethanolic extract, 8 mL of 10^−^² M silver nitrate (AgNO₃) solution was added to a labeled beaker containing the ethanolic extract, yielding a 5:4 volume ratio. The mixture was incubated in an orbital shaker (IST3075R) at 45°C and 150 rpm for 24 hours. This procedure was adapted and optimized based on previously reported protocols to improve the synthesis conditions. The resulting AgNP extract was protected from light in amber bottles and oven-dried to calculate dry yield (powder form).

#### Gel formulation

The dried AgNP-ethanolic extract was incorporated into a 1% hydroxyethyl cellulose gel matrix. A 1:1 ratio of powder (mg) to gel (mL) was used. The components were homogenized in a sterile beaker using magnetic stirring combined with ultrasonic agitation. The final formulation was stored in sterile 5 mL syringes under aseptic conditions.

#### Antimicrobial assays

Microbial strain and culture conditions

The antimicrobial activity was tested against *E. faecalis* (ATCC 29212). Frozen stocks were reactivated in Tryptic Soy Broth (TSB) and incubated at 37°C for 24 hours under aerobic conditions in a bacteriological incubator. The inoculum was standardized to the 0.5 McFarland scale (~1.5 × 108 colony-forming unit [CFU]/mL) using spectrophotometric adjustment at 600 nm.

Experimental groups and controls

Negative control: 1% hydroxyethyl cellulose gel with bacterial inoculum.

Positive controls and test groups: 1% hydroxyethyl cellulose containing crude extract, nanoparticulated extract, 2.5% NaOCl gel, and 2% CHX digluconate gel obtained from a pharmaceutical compounding pharmacy, all with standardized *E. faecalis* inoculum.

CFU/mL quantification

The CFU assay was conducted using the macrodilution method in BHI broth [19]. Each test tube received 2.5 mL BHI and 50 μL of bacterial inoculum. After pre-incubation and contamination check via Petri plating, 2.5 mL of each test/control substance was added. Serial dilutions (10^−^¹ and 10^−^²) were prepared and exposed to the test substances for 2 hours at 37°C, in order to evaluate the short-term antibacterial effect. Subsequently, 20 μL from each dilution was plated on Mueller-Hinton agar and incubated for 24 hours. CFUs were subsequently counted on plates presenting 30–300 colonies, and results were expressed as CFU/mL after correction by the dilution factor.

All antimicrobial assays were performed in triplicate independent experiments to ensure reproducibility. CFU/mL were calculated by counting visible colonies on agar plates and multiplying by the corresponding dilution factor. The percentage reduction in bacterial load was calculated by comparing the initial CFU/mL values with those obtained after exposure to the tested substances.

Agar well diffusion test

Mueller-Hinton agar plates (90 mm, 4 mm depth) were inoculated with 20 μL of standardized *E. faecalis* suspension and allowed to dry at 37°C for 15 minutes. Wells (9 mm diameter) were bored into the agar and filled with 30 μL of each gel formulation. Plates were pre-diffused for 2 hours at room temperature and incubated at 37°C for 24 hours. Antimicrobial activity was assessed by measuring the diameter of growth inhibition zones using two methods:

Technique I: Horizontal and vertical diameters averaged.

Technique II: Four diagonal measurements (X-shape) averaged.

Minimum inhibitory concentration – resazurin assay

Minimum inhibitory concentration (MIC) values were determined by broth microdilution. In 96-well plates, 90 μL of Mueller-Hinton broth, 10 μL of inoculum, and 0.2 mL of extract or controls were added per well. The extract was prepared at an initial concentration of 100 mg/mL, corresponding to 20 mg per 0.2 mL aliquot (defined as 100% concentration). Serial two-fold dilutions of the extract were then performed, resulting in final concentrations of 100% (20 mg), 50% (10 mg), 25% (5 mg), and 12.5% (2.5 mg).

Serial twofold dilutions of the test compounds were prepared in 96-well microtiter plates. The microdilution plates were then incubated at 37°C for 18–24 h. After incubation, 20 μL of 0.01% aqueous resazurin solution was added to each well and plates were further incubated at 37°C for 1–4 h. Resazurin acts as a redox indicator that is reduced by metabolically active bacterial cells, producing a color change from blue (oxidized form) to pink (resorufin). The MIC was defined as the lowest concentration of the extract that prevented this color change, indicating inhibition of bacterial metabolic activity [21].

Growth control wells containing broth and bacterial inoculum without test compounds were included to confirm bacterial viability. Sterility control wells containing only broth were used to verify medium sterility. In addition, solvent control wells containing the extract vehicle were included to exclude potential interference of the solvent with bacterial growth or with the resazurin reaction.

### Statistical analysis

Data were compiled and analyzed using the R statistical software (version 4.3.0). The distribution of continuous variables was assessed for normality using the Shapiro–Wilk test. For CFU/mL counts, comparisons between initial and final bacterial loads within each treatment group were performed using the Wilcoxon signed-rank test, since these measurements represent paired observations. Statistical significance was established at *p* < 0.05. For inhibition zone measurements, results from the pilot screening assay were reported as absolute values in millimeters. In the agar well diffusion assay, inhibition zones were expressed as mean ± standard deviation based on triplicate measurements obtained using two measurement techniques. These results were analyzed descriptively to compare antibacterial performance among the tested substances. The MIC was determined qualitatively as the lowest concentration preventing the color change in the resazurin assay and therefore did not involve inferential statistical testing.

All experiments were performed in triplicate independent assays (*n* = 3). Given the exploratory design of the study, no formal sample size calculation or effect size estimation was performed.

## Results

### Preliminary evaluation of extract characteristics

#### Yield of extract production

Following the extraction procedures, three types of extracts – hydroalcoholic, alcoholic, and shaker-assisted alcoholic – were evaluated in terms of liquid volume (mL) and crude yield by weight (mg). The hydroalcoholic and alcoholic extracts both resulted in 400 mL of liquid extract, yielding 1.900 g and 1.1142 g of dried crude extract, respectively. In contrast, the shaker-assisted alcoholic extraction produced a lower liquid volume of 280 mL but yielded the highest amount of crude extract at 2.0945 g. After volume measurements, all samples were subjected to drying in a circulating air oven, and the resulting solid residues were weighed using a precision analytical balance. Notably, the shaker-assisted ethanolic method, despite yielding a smaller liquid volume, resulted in the highest dry mass recovery, indicating its greater extraction efficiency compared to the other methods.

#### Yield of nanoparticle extract production

The nanoparticulated extract of *C. citratus* was produced using a shaker-assisted ethanolic extraction process. The final volume and dry mass of the nanoparticulate formulation were determined, resulting in a yield of 360 mL and a crude dry mass of 1.260 mg. These values indicate a lower overall yield when compared to non-nanoparticulated extracts.

#### pH analysis of the extracts

pH measurements of the various extract types (hydroalcoholic, alcoholic, aqueous, and shaker-assisted ethanolic) collected during different months (June and July) revealed minimal variation. The extracts showed slightly acidic pH values ranging from 4 to 6, indicating that harvest season had no significant impact on the pH profile (Table 1). Based on these findings, samples from the same extraction method were pooled and stored in amber flasks for further use.

**Table 1:** pH values of lemongrass (*C. citratus*) extracts according to extraction method, plant harvest month (June or July), and extract concentration (mg/mL).

Extract type	Concentration (mg/mL)	pH (June / July)
June Hydroalcoholic	200	5
June Hydroalcoholic	100	4
July Hydroalcoholic	200	6
July Hydroalcoholic	100	6
June Alcoholic	200	6
July Alcoholic	100	5
June Aqueous	100	6
July Aqueous	200	6
Shaker-assisted Alcoholic	200	4
Shaker-assisted Alcoholic	100	4

Hydroalcoholic, alcoholic, aqueous, and shaker-assisted alcoholic extraction methods were evaluated to characterize the physicochemical properties of the extracts used in the antimicrobial assays.

#### Inhibition zone in preliminary agar diffusion test

Agar diffusion tests showed minimal variation among extracts of the same type. However, the shaker-assisted ethanolic extract demonstrated superior inhibition compared to both other extracts and the 2.5% NaOCl control (Table 2). This extract produced an inhibition zone in the preliminary agar diffusion test 17.55% larger than NaOCl and showed no significant difference between harvest months. Thus, it was selected as the optimal formulation for further experimentation in its natural and nanoparticulated forms.

**Table 2:** Inhibition zone diameters (mm) produced by different *C. citratus* extracts and control substances against *E. faecalis* in the pilot antimicrobial screening using the agar diffusion method.

Extract type / Substance	Inhibition zone (mm)
Hydroalcoholic (June 200 mg/mL)	11.57
Hydroalcoholic (June 100 mg/mL)	11.87
Hydroalcoholic (July 200 mg/mL)	11.17
Hydroalcoholic (July 100 mg/mL)	11.05
Alcoholic (June 200 mg/mL)	11.91
Alcoholic (July 100 mg/mL)	10.77
Aqueous (June 100 mg/mL)	0
Aqueous (July 200 mg/mL)	0
Shaker-assisted Alcoholic (June 200 mg/mL)	14.34
Shaker-assisted Alcoholic (June 100 mg/mL)	14.87
Sodium Hypochlorite 2.5%	12.42
Chlorhexidine 2%	25.06

Sodium hypochlorite (2.5%) and chlorhexidine (2%) were used as positive controls.

#### Antimicrobial activity of natural and nanoparticulated extracts

Antimicrobial activity and MIC

Both the shaker-assisted ethanolic extract (natural form) and its nanoparticulated counterpart inhibited bacterial growth at higher concentrations; however, the nanoparticulate formulation maintained inhibitory activity at the 50% dilution of the initial extract concentration, indicating that the nanoparticulated formulation required a lower effective dose than the natural extract to inhibit bacterial growth.

CFU/mL

Quantification of CFUs revealed reductions of 63.61% with the natural extract and 69.9% with the nanoparticulated formulation. Both extracts showed greater antibacterial activity than the 2.5% NaOCl control (49.95% reduction), while 2% CHX exhibited the highest antibacterial efficacy, with a 99.24% reduction in CFU/mL. CHX differed significantly from all other groups (*p* < 0.05). Both the natural and nanoparticulated extracts also showed significantly greater reductions than NaOCl and the negative control. No significant difference was observed between the natural and nanoparticulated extracts; however, the nanoparticulated formulation showed a slightly higher reduction in CFU counts. In contrast, the 1% Natrosol negative control showed an increase in bacterial growth (Table 3).

**Table 3:** Antibacterial activity against *E. faecalis* expressed as colony-forming units per milliliter (CFU/mL) before and after treatment with the tested formulations (dilution 10^−^¹).

Substance	Initial CFU/mL	Final CFU/mL	*P*-value
Natural Lemongrass Extract + Natrosol 1%	2539	924	< 0.05*
Nanoparticulated Lemongrass Extract + AgNO^3^ + Natrosol 1%	3184	956	
Sodium Hypochlorite 2.5% Gel	3708	1856	
Chlorhexidine Digluconate 2% Gel *	2238	17	
Natrosol 1% Gel (negative control)	1128	22,280	

Data represent bacterial counts obtained in the microbiological assay.

* Wilcoxon signed-rank test. Data obtained from triplicate experiments (*n* = 3). CFU: colony-forming unit.

Agar well diffusion assay

The nanoparticulated lemongrass extract produced an inhibition zone 21.65% larger than that of the natural extract after 24 hours of incubation. Both extracts produced smaller inhibition zones than the 2.5% NaOCl gel, while CHX 2% showed intermediate values. No significant differences were observed between the nanoparticulated extract and 2% CHX in either measurement technique (Table 4), indicating comparable antibacterial performance against *E. faecalis*. As expected, the 1% Natrosol negative control showed no inhibition.

**Table 4:** Mean inhibition zone diameters (mm ± standard deviation) produced by the tested formulations against *E. faecalis*, measured using two agar diffusion techniques (Technique I and Technique II).

Substance	Technique I (Mean ± SD)	Technique II (Mean ± SD)	Combined I + II (Mean ± SD)
Natural Extract + Natrosol 1%	9.05 ± 0.57	4.37 ± 1.05	6.71 ± 2.49
Nanoparticulated Extract + Natrosol 1%	11.55 ± 6.55	6.75 ± 3.88	9.15 ± 5.89
Sodium Hypochlorite 2.5% Gel	25.74 ± 3.40	13.86 ± 4.71	19.80 ± 7.22
Chlorhexidine Digluconate 2% Gel	17.95 ± 4.51	8.81 ± 1.94	13.38 ± 5.74
Natrosol 1% Gel	0.00 ± 0.00	0.00 ± 0.00	0.00 ± 0.00

The combined value represents the overall mean of both techniques. Data obtained from triplicate experiments (*n* = 3).

## Discussion

CHAS are essential because endodontic instruments cannot reach the full complexity of the root canal system [22]. Consequently, there is a need to explore alternatives that meet the ideal criteria for a CHAS while maintaining comparable efficacy to conventional substances [3]. Based on this rationale, the present study aimed to evaluate the antimicrobial potential of a nanoparticulated *C. citratus* gel extract against *E. faecalis*.

The therapeutic effects of *C. citratus* are mainly attributed to its rich phytochemical composition, which includes citral, geraniol, flavonoids, and alkaloids [7]. Studies have demonstrated its antibacterial potential against pathogens associated with dental caries, supporting its role as a potential candidate pending further validation in endodontic disinfection [9]; however, its efficacy against *E. faecalis* still requires further investigation.

Scientific evidence indicates that the resistance of microorganisms like *E. faecalis* to conventional CHAS, coupled with the complexity of the root canal system (RCS), contributes to endodontic treatment failure [13]. This justifies the need to develop new CHAS capable of effectively eliminating resistant bacteria, which represents a biotechnological advancement.

The combination of the plant extract with nanoparticles enabled a ‘green synthesis’ approach, offering an eco-friendly and cost-effective alternative for nanoparticle production [21]. In this process, hydrophilic metabolites and flavonoids act as natural stabilizers and protectants, which may enhance the bioactivity of the resulting nanoparticles [20]. Notably, the nanoparticulated formulation demonstrated superior antimicrobial efficacy compared to the unformulated extract, showing relevant antibacterial activity in comparison with conventional control agents. These findings suggest that nanoparticle incorporation not only preserves the intrinsic properties of the plant extract but may also potentiate its antimicrobial action, supporting the potential of such formulations in endodontic applications [23, 24].

Silver nanoparticles are known to possess intrinsic antimicrobial activity, which may have contributed to the enhanced antibacterial effect observed in the nanoparticulated formulation [18, 19]. In this study, nanoparticles were produced through plant-mediated green synthesis using *C. citratus*, suggesting that the observed activity may result from the combined effect of silver nanoparticles and plant-derived compounds. Future studies including control formulations containing silver nanoparticles without plant extract may help clarify the relative contribution of each component.

From a clinical perspective, these findings suggest that nanoparticulated plant-based formulations may represent a potential adjunct or alternative to conventional irrigants; however, their effectiveness under clinically relevant conditions, such as within dentinal tubules, biofilm structures, and in vivo environments, still requires further investigation.

Various extraction techniques exist for producing plant-based derivatives, including maceration, infusion, and ultrasonic-assisted extraction [7]. In our pilot study, alcoholic extracts yielded better results than hydroalcoholic ones when both were produced using ultrasound. However, the shaker-assisted alcoholic extract, although yielding a lower extract volume, demonstrated superior antibacterial activity [24].

Previous studies have indicated that extract yield can be influenced by the harvest period [10]. Although this was not the primary focus of the present work, our observations suggested that samples collected in June produced a higher dry extract yield than those harvested in July, despite similar cultivation and environmental conditions [26, 27]. These findings highlight the potential impact of seasonal factors on extract productivity and underscore the need for further comparative studies to better understand how harvest timing may affect both yield and bioactive compound content.

Gels have lower flowability than solutions due to their molecular weight, which may reduce the inhibition zone. To ensure standardization during agar diffusion tests, all CHAS formulations were adjusted to the same molecular weight using 1% hydroxyethyl cellulose gel [28, 29]; however, it should be considered that physicochemical factors such as viscosity may still influence diffusion behavior and, consequently, inhibition zone measurements. Results showed superior inhibition zones for the nanoparticulated extract compared to its natural counterpart, indicating enhanced antimicrobial efficacy against *E. faecalis*.

Previous studies have reported that the antimicrobial activity of lemongrass extracts against other bacterial species may vary depending on the extraction solvent, with alcoholic extracts generally demonstrating greater efficacy than aqueous preparations [30–32]. Similar antimicrobial effects against *E. faecalis* have also been reported in studies evaluating essential oils and plant-derived compounds for endodontic applications [8, 9].

While single-method testing may reveal antimicrobial activity, combining methods offers a more comprehensive analysis [33]. This study used agar well diffusion, CFU counts, and MIC determination to verify antibacterial efficacy. Macrodilution with CFU counting revealed a significant reduction in bacterial load for both extract forms, with a greater reduction in the nanoparticulated extract.

MIC testing confirmed effective inhibition at low concentrations, with bacterial growth inhibition observed at 50% of the initial extract concentration for the nanoparticulated extract, further supporting its antibacterial potential. Overall, this study presents a novel biotechnological advance for potential endodontic applications although it is limited by a lack of comparative literature. Additional studies are required to explore the best harvest conditions, extract yields, and a broader antibacterial spectrum.

It is important to acknowledge that comprehensive physicochemical characterization of nanoparticles, including particle size distribution, morphology, zeta potential, and stability assessment, plays a fundamental role in understanding nanomaterial behavior and ensuring reproducibility. In the present study, the primary focus was the preliminary biological evaluation of the antibacterial activity of the proposed formulation. Therefore, additional physicochemical characterization analyses will be addressed in future investigations to further elucidate the structural properties and stability of the nanoparticle system.

In addition, some experimental limitations should be considered when interpreting the present findings. The antibacterial evaluation was performed using planktonic bacterial cultures and did not include biofilm or dentin substrate models, which more closely simulate clinical conditions of the root canal system. Furthermore, cytotoxicity assays, tissue dissolution capacity, and substantivity tests were not evaluated in this preliminary investigation. These aspects are important for determining the clinical safety and functional performance of potential chemical auxiliary substances and should be addressed in future studies.

Finally, these findings support the use of lemongrass as a promising alternative antibacterial agent for endodontic treatment. Compared to conventional CHAS, plant-based therapies offer benefits such as lower toxicity, reduced effective dosages, activity against resistant pathogens (e.g. *E. faecalis*), and low-cost production methods. However, further studies are required to clarify its mechanisms of action, ideal cultivation conditions, harvest timing, extract yield, and efficacy against a broader range of microorganisms, as well as to confirm its clinical applicability.

## Conclusion

This study demonstrated that *C. citratus* exhibits antimicrobial activity against *E. faecalis*. The alcoholic extraction method yielded superior antibacterial effects. The nanoparticulated *C. citratus* extract, formulated with 1% hydroxyethyl cellulose, exhibited significantly greater bacterial inhibition than the natural extract and demonstrated relevant antibacterial activity, although not exceeding the performance of CHX digluconate, supporting the potential of nanoparticle-enhanced formulations as antimicrobials.

## Data Availability

The data supporting the findings of this study are included in the article, and additional datasets are available from the corresponding author upon reasonable request.

## Significance of the work

This study demonstrates that nanoparticle-based formulations of *Cymbopogon citratus* significantly enhance antibacterial activity against *Enterococcus faecalis*, a clinically relevant pathogen associated with persistent endodontic infections. By combining phytotherapeutic compounds with nanoparticle technology and gel-based delivery, the findings highlight a material-oriented strategy capable of improving antimicrobial performance without increasing formulation complexity.

The nanoparticulate gel showed superior antibacterial efficacy compared with the non-nanoparticulated extract, achieving bacterial reduction comparable to chlorhexidine and superior to NaOCl in CFU-based assays. These results emphasize the role of nanoparticle incorporation as a key factor in potentiating the antimicrobial activity of plant-derived compounds.

By integrating green synthesis, phytochemistry, and nanotechnology, this work contributes to the development of innovative dental biomaterials with potential application in endodontic disinfection. The study provides relevant experimental evidence for researchers seeking alternative, sustainable approaches to enhance antimicrobial efficacy in endodontic materials.

## Reporting guidelines

This *in vitro* experimental study was conducted in accordance with established methodological standards for antimicrobial testing. Access to genetic heritage was duly registered in the Brazilian National System for the Management of Genetic Heritage and Associated Traditional Knowledge (SisGen), under registration number A75757E.

## Permission to reproduce material

No previously published material (figures, tables, or text) requiring permission was reproduced in this manuscript.

## Clinical trial registration

Not applicable. This study is not a clinical trial.
